# Reducing mitochondrial dysfunction through combination therapy to limit ischemia-reperfusion injury in male DCD rats

**DOI:** 10.3389/fcvm.2025.1625385

**Published:** 2026-01-21

**Authors:** Zachary Kiernan, Gina Labate, Qun Chen, Mohammed Quader

**Affiliations:** 1 Division of Cardiothoracic Surgery, Department of Surgery, Virginia Commonwealth University, Richmond, VA, United States; 2 Division of Cardiology, Department of Internal Medicine, Virginia Commonwealth University, Richmond, VA, United States; 3 Pauley Heart Center, Virginia Commonwealth University Health System, Richmond, VA, United States; 4 Surgical Service, Department of Surgery, Central VA, United States VA Healthcare System, Richmond, VA, United States

**Keywords:** cyclosporine A, donation after circulatory death, heart failure, MDL-28170, MPTP

## Abstract

**Introduction:**

Two predominant pathways contribute to ischemia reperfusion injury (IRI) following donation after circulatory death (DCD): mitochondrial permeability transition pore (MPTP) opening and Calpain-1 (CPN1) activation. Each pathway has established inhibitors; Cyclosporine A (CyA) and MDL-28170 (MDL), respectively, which are effective in modulating IRI in a DCD heart with 25 min of warm ischemia time (WIT). We studied the effect of co-administering CyA and MDL during reperfusion on infarct size and graft function in DCD rat hearts with extended WIT of 35 min.

**Methods:**

Male rats were exposed to 35 min of warm ischemia followed by 90 min of reperfusion. During reperfusion, hearts were given either 0.5 mM of CyA, 10 mM of MDL, or mixed CyA and MDL. Cardiac function and coronary flow rates were monitored throughout reperfusion and infarct size at the end of reperfusion.

**Results:**

Infarct size in hearts treated with mixed CyA + MDL (31.59 ± 7.1%) was less than that of MDL-treated hearts (33.26 ± 4.3%) but larger than CyA-treated hearts (25.49 ± 5.9%). Graft function and coronary flow rates were variable amongst groups. CyA-treated hearts had more profound infarct size reduction when compared to MDL, and no additional synergistic effect was seen with combination treatment.

**Discussion:**

Our results indicate that MPTP opening contributes significantly to the development of IRI in DCD hearts.

## 1 Introduction

Heart transplantation remains the “gold standard” treatment for patients with advanced, medically refractory heart failure. There is a persistent shortage of suitable hearts for transplantation. In 2022, the total number of patients awaiting transplantation was 7,519. This represents a 28% increase from 5,869 patients in 2011 (1). Donation after circulatory death (DCD) donor hearts have been increasingly used in the past decade, but the obligatory ischemia-reperfusion injury (IRI) that occurs with the DCD donation process, resulted in primary graft dysfunction in up to 45% of recipients (2). Concerns surrounding PGD have limited the warm ischemia time (WIT) to <30 min, thus only 19.7% of consented DCD hearts for transplantation were utilized (3–6). Since IRI is proportional to the duration of WIT, prolonged WIT (>30 min) was cited as the most common reasons for organ refusal.

While the mechanisms behind IRI are multi-factorial, two key pathways exist. The first is the activation of calcium-activated cysteine proteases, known as Calpains, found within the mitochondria and cytosol of cells (7). Calpain 1 (CPN1) and Calpain 2 (CPN2) are the two predominant isoforms and, when activated, they cleave both cytosolic and mitochondrial proteins. This leads to profound mitochondrial dysfunction through the generation of reactive oxygen species, reduction in adenosine triphosphate (ATP) production, and the release of proapoptotic proteins, ultimately resulting in cardiomyocyte cell death (8–10).

The second major pathway involves opening of the mitochondrial permeability transition pore (MPTP). The MPTP is a non-selective pore found within the inner mitochondrial membrane (IMM) and is typically closed, even during periods of ischemia (11). Upon reperfusion high concentrations of intracellular Ca^2+^ and the rapid normalization of cellular pH, results in the MPTP opening leading to the electron transport chain (ETC) uncoupling, mitochondrial swelling, and massive ionic shifts resulting in cell rupture. In addition, proapoptotic Cytochrome *c*, which activates Caspase-3 and apoptosis inducing factor (AIF) are released ultimately leading to cell death (12–14).

Both CPN1/CPN2 and MPTP opening have known inhibitors; MDL-28170 (MDL) and Cyclosporine A (CyA), respectively. Previous work done by our lab has shown that at a dose of 10 μM MDL significantly reduced calpain activity compared to vehicle or 3 μM MDL (15). Furthermore, in a DCD rat heart, the administration of 10 μM of MDL, given throughout a period of ex-vivo reperfusion, significantly limited calpain activation, reduced infarct size and improved mitochondrial function, both oxidative phosphorylation (OXPHOS) and calcium retention capacity (CRC) (8, 16). Similarly we also demonstrate that 0.5 μM of CyA, administered for the first 15 min of a 90 min reperfusion period, reduced infarct size in DCD hearts and also resulted in significant improvement in OXPHOS and CRC function (17, 18). Our work on DCD hearts with CyA added to the already large body of work supporting the use of CyA to mitigate ISI in cardiomyocytes (19).

Our prior work on DCD heart IRI modulation was done with WIT of 25 min, simulating current clinical DCD heart transplantation practice. Given the promising results of using CyA and MDL individually to limit IRI in DCD hearts with 25 min of WIT, we explored whether administering CyA and MDL would have a synergistic response in a DCD heart with prolonged WIT. Our objective is to study the modulation of IRI with combination of CyA and MDL in a DCD heart with WIT of 35 min.

## 2 Materials and methods

All experimental animals were cared for in accordance with institutional guidelines and the *Guide for the Care and Use of Laboratory Animals* prepared by the Institute of Laboratory Animal Resources (20). The study protocol was approved by the Institutional Animal Care and Use Committee of Virginia Commonwealth University (protocol number AD10002961) and the Central Virginia VA Health Care System (protocol number 02253). Animals were obtained through Envigo (Inotiv, Inc) and were housed two per cage within a temperature and humidity-controlled vivarium with alternating 12-h light and dark cycles. The animals had continuous access to water and food (Inotiv Teklad LM-485 rat diet).

### 2.1 Preparation of rat hearts for ischemia and reperfusion

**Figure 1:**
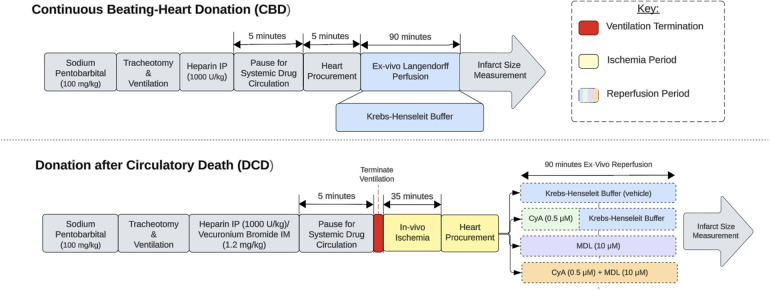
Experimental design. Experimental design comparing continuous beating-heart donation (CBD) and donation after circulatory death (DCD) procedures. The red section indicates ventilator termination, which initiates the ischemic period. The yellow sections represent *in-vivo* ischemia periods, while the blue sections represent *ex-vivo* reperfusion on the Langendorff apparatus. The reperfusion period for DCD hearts is divided into 5 categories based on the treatment paradigm used.

Male Sprague-Dawley rats, weighing approximately 350–370 g, were separated into one of five groups based on their allotted ischemia time and treatment protocol (Table 1). Donor rats were anesthetized with an intraperitoneal injection of pentobarbital (100 mg/kg) (Sagent Pharmaceuticals, #0676-20). Once fully anesthetized, the neck was incised to expose the trachea. A small tracheotomy was made and a 14 g angio-catheter was inserted into the tracheal opening. This was connected to a small animal ventilator (RoVent® Advanced Small Animal Ventilator, Kent Scientific Corporation, Torrington, CT, USA) set to deliver a tidal volume of 2.65 mL at 70 breaths per minute. Next, electrocardiogram electrodes (AD Instruments, Colorado Springs, CO, USA) were placed to continuously monitor the animals heart rate using an ADI Bio Amp and LabChart software (AD Instruments, Colorado Springs, CO, USA). Next, intraperitoneal heparin (1,000 U/kg) (Fresenius Kabi, #403577) and intramuscular vecuronium bromide (1.2 mg/kg) (Sigma-Aldrich, #76904) were administered for systemic anticoagulation and muscle paralysis, respectively. After 5-min ventilator support was terminated, thereby marking the start of the 35-min of warm ischemia period. The time from ventilatory termination to cardiac arrest was typically between 9 and 12 min, consistent with our previously published data (21). At approximately 33 min of ischemia time, the heart procurement process was started to allow enough time for placement of the heart on the Langendorff apparatus. Hearts from continuous beating-heart donors (CBD) were procured without ventilator termination or ischemia and served as a control (Figure 1).

**Table 1:** Experimental groups.

Groups	Experimental protocol	Sample size
CBD	Without *in vivo* ischemia, hearts received 90 min of continuous K-H buffer perfusion from 2 L reservoir	*N* = 10
DCD + Vehicle	After 35 min of *in vivo* ischemia, hearts received 90 min of continuous K-H buffer perfusion from 2 L reservoir	*N* = 7
DCD + CyA only	After 35 min of *in vivo* ischemia, hearts received 15 min of 0.5 μM CyA in K-H buffer from 500 mL reservoir, followed by 75 min of perfusion with K-H buffer alone from 1,500 mL reservoir	*N* = 10
DCD + MDL only	After 35 min of *in vivo* ischemia, hearts were perfused with 10 μM MDL in K-H buffer for 90 min from 2 L reservoir	*N* = 8
DCD + CyA + MDL mixed	After 35 min of *in vivo* ischemia, hearts received a mixture of 0.5 μM CyA and 10 μM MDL mixed in 500 mL K-H buffer for the initial 15 min, followed by perfusion with 10 μM MDL alone in 1,500 mL K-H buffer for an additional 75 min, making a total MDL perfusion time of 90 min	*N* = 8

CBD, continue beating-heart donation; DCD, donation after circulatory death; Min, minute; *N* represents individual animals; CyA, cyclosporine A.

### 2.2 Procedure for heart reperfusion and treatment

Once procured, hearts were mounted on a Langendorff apparatus and perfused at a constant pressure of 73 mmHg for 90 min. A small incision was made in the left atrium to insert a balloon catheter into the left ventricle. The catheter was connected to a physiological pressure transducer (AD Instruments, Colorado Springs, CO, USA) to continuously record left ventricular developed pressure (LVDP) using LabChart software (AD Instruments, Colorado Springs, CO, USA). Hearts were perfused with modified Krebs-Henseleit (K-H) buffer [115 mM NaCl, 4.0 mM KCl, 2.5 mM CaCl2, 26 mM NaHCO3, 1.1 mM MgSO4, 0.9 mM KH2PO4, 5.5 mM glucose, and 5 IU/L regular insulin (Novo Nordisk Inc, #1833-11)] at 37 °C. The buffer was continuously oxygenated with 95% O2% and 5% CO2 to maintain a pH of 7.4. The experimental protocols for each group of animals are as follows:

**CBD hearts:** Without *in vivo* ischemia, hearts received 90 min of continuous K-H buffer perfusion from 2 L reservoir.
**Vehicle hearts:** After 35 min of *in vivo* ischemia, hearts received 90 min of continuous K-H buffer perfusion from 2 L reservoir.
**CyA** (Sigma-Aldrich, #C3662)**:** After 35 min of *in vivo* ischemia, hearts received 15 min of 0.5 μM CyA in K-H buffer from 500 mL reservoir, followed by 75 min of perfusion with K-H buffer alone from 1,500 mL reservoir.
**MDL** (Sigma-Aldrich, #M6690): After 35 min of *in vivo* ischemia, hearts were perfused with 10 μM MDL in K-H buffer for 90 min from 2 L reservoir.
**CyA + MDL mixed:** After 35 min of *in vivo* ischemia, hearts received a mixture of 0.5 μM CyA and 10 μM MDL mixed in 500 mL K-H buffer for the initial 15 min, followed by perfusion with 10 μM MDL alone in 1,500 mL K-H buffer for an additional 75 min, making a total MDL perfusion time of 90 min.

Heart rate and LVDP were recorded at 15, 30, 45, 60, and 90 min of reperfusion. At the same time points, coronary effluent was collected to measure volume, and aliquots were stored at −80 °C for enzyme activity analysis. At the end of the 90-min reperfusion period, hearts were removed from the Langendorff apparatus, wrapped in foil, and stored at −20 °C for infarct size measurement.

### 2.3 Procedure for infarct size measurement

Frozen hearts were carefully sectioned along the long axis into four pieces, each approximately 2–3 mm thick. Sections were incubated in the 1% TTC solution at 37 °C for 20 min, then transferred to 10% formalin at 4 °C for ∼24 h to enhance tissue fixation. After formalin fixation, sections were dried, individually weighed, and placed between two clear plastic sheets for digital scanning. Images were analyzed using ImageJ software (National Institutes of Health, Bethesda, MD, USA) to determine the percentage of infarcted tissue by weight.

### 2.4 Statistical analysis

Data was analyzed using GraphPad Prism 10 Software (Boston, MA, USA). Normality of the data was assessed using the Shapiro–Wilk test. Comparisons of means for infarct size and functional data between groups were made using a one-way Analysis of Variance (ANOVA) with *post-hoc* Bonferroni correction if data passed the normality and equal distribution test. Data that failed the test of normality or equal distribution test were compared using a non-parametric Kruskal–Wallis one-way analysis of variance (ANOVA) on ranks followed by Dunn's analysis for multiple groups. A significance level of *α* < 0.05 was established *a priori* to analysis and all data are reported as mean ± standard deviation (SD).

## 3 Results

### 3.1 Infarct size reduction is more with CyA compared to MDL

**Figure 2:**
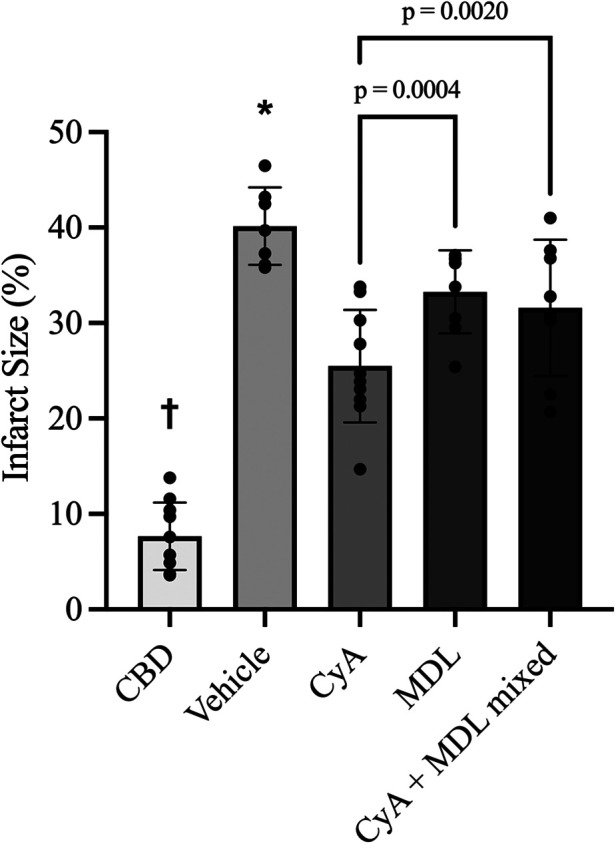
Infarct size measurements across experimental groups as measured using triphenyL–tetrazolium chloride (TTC) staining and digital planimetry. Effects of MDL-28170 (MDL), cyclosporine A (CyA) and combination treatment on infarct size in DCD rat hearts following 35 min of ischemia. Infarct size in DCD hearts treated with CyA (25.49 ± 5.90%), MDL (33.26 ± 4.35%) and combination CyA + MDL (31.59 ± 7.14%) were significantly lower than vehicle hearts (40.16 ± 4.06%). Infarct size in hearts treated with mixed CyA + MDL was lower than that of MDL alone, but no statistically significant difference was noted. Furthermore, combination treatment did not exceed the infarct size reduction produced by CyA treatment alone. One-way analysis of variance used for statistical analysis. All data expressed as mean ± SD. † Represents statistical significance (*p* < 0.05) between CBD hearts and all other groups. * Represents statistical significance (*p* < 0.05) between vehicle hearts and all other groups.

TTC staining showed that CBD hearts without ischemia had the smallest infarct size (7.67 ± 3.53%), whereas vehicle treated hearts subjected to 35 min of ischemia had the largest infarct size (40.16 ± 4.06%). Compared to the vehicle group, infarct size was significantly reduced by CyA (25.49 ± 5.90%), MDL (33.26 ± 4.35%) and combination CyA + MDL (31.59 ± 7.14%) treatments. Moreover, infarct size in CyA-treated hearts was significantly lower than in MDL-treated hearts (Figure 2).

### 3.2 CyA and MDL combination had marginal improvement in infarct size reduction

Infarct size in the CyA + MDL group (31.59 ± 7.14%) was significantly smaller than in vehicle hearts (40.16 ± 4.06%). However, the combination treatment did not reduce infarct size beyond that observed with CyA alone (Figure 2).

### 3.3 Limited synergistic effect of CyA and MDL combination on improving DCD heart function

**Figure 3:**
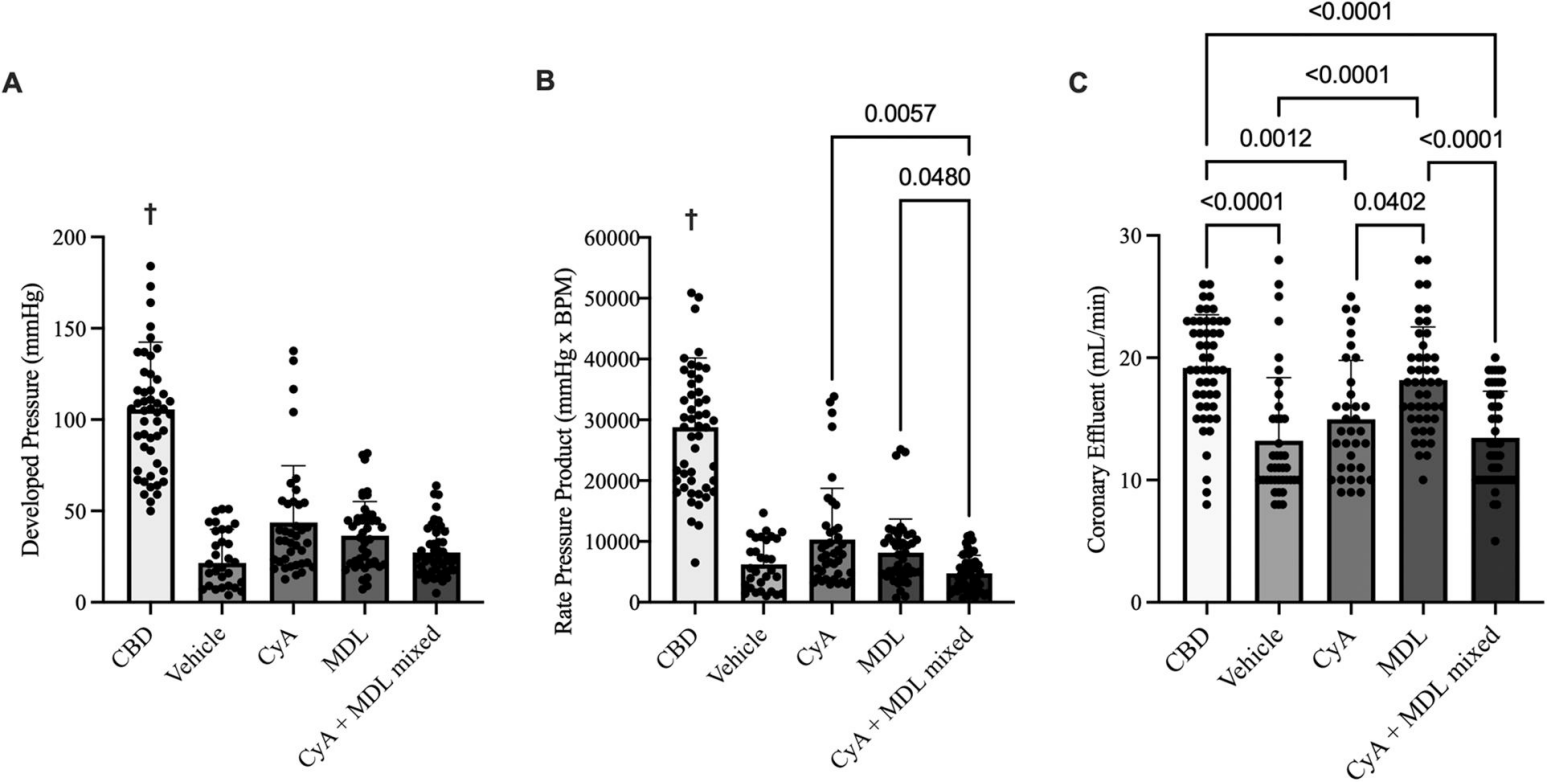
Comparison of aggregated left ventricular developed pressure (LVDP) **(A)**, rate pressure product (RPP) **(B)**, and coronary effluent **(C)** across experimental groups. A balloon catheter connected to a pressure transducer was placed into the left ventricle to record LVDP and RPP throughout the 90 min of reperfusion. Of the treated hearts, LVDP was highest in the CyA group (43.71 ± 4.97 mmHg) and lowest in the mixed CyA + MDL group (27.22 ± 13.45 mmHg). No differences were noted between the mixed CyA + MDL group and CyA-treated (43.71 ± 31.05 mmHg) or MDL treated (36.52 ± 18.61 mmHg) hearts. Of the treated hearts, RPP was highest in the CyA-treated hearts (10,332 ± 8,417 mmHg*BPM) and lowest in the mixed CyA + MDL group (4,747 ± 2,965 mmHg*BPM). Coronary effluent was collected for 1 min at 15, 30, 45, 60 and 90 min of reperfusion. Flow rates varied between groups, with the highest rate in the MDL-treated hearts (18.18 ± 4.35 mL/min) and lowest in the mixed CyA + MDL group (13.46 ± 3.81 mL/min). One-way analysis of variance used for statistical analysis. All data expressed as mean ± SD. † Represents statistical significance (*p* < 0.05) between CBD hearts and all other groups.

Heart function was recorded continuously throughout reperfusion, with measurements taken at 15, 30, 45, 60, and 90 min. These values were averaged for each group to generate aggregate LVDP and RPP. Overall, LVDP was highest in CBD hearts (105.60 ± 36.83 mmHg) and lowest in vehicle hearts (24.73 ± 15.49 mmHg). Among the treated hearts, LVDP was highest in the CyA group (43.71 ± 31.05 mmHg), followed by the MDL group (36.52 ± 18.61 mmHg), and the mixed CyA + MDL group (27.22 ± 13.45 mmHg) (Figure 3A).

Across all groups, RPP (mmHg·BPM) was highest in CBD hearts (28,816 ± 11,348) and lowest in vehicle hearts (6,245 ± 4,045). Among the treatment groups, RPP was highest in CyA-treated hearts (10,332 ± 8,417), followed by the MDL group (8,174 ± 5,507), and the CyA + MDL group (4,747 ± 2,965) (Figure 3B).

Coronary effluent was collected for 1 min at 15, 30, 45, 60, and 90 min of reperfusion to determine flow rate, and the values were averaged for each group. Flow rate was highest in CBD hearts (19.18 ± 4.35 mL/min) and lowest in vehicle hearts (13.23 ± 5.15 mL/min). Among the treated hearts, flow rate was highest in the MDL group (19.18 ± 4.35 mL/min), followed by the CyA group (14.97 ± 4.81 mL/min), and the mixed CyA + MDL group (13.46 ± 3.81 mL/min). Significant differences were observed between CyA- and MDL-treated hearts, as well as between MDL-treated and combination-treated hearts (Figure 3C).

## 4 Discussion

The first human heart transplant, performed by Christiaan Barnard in 1967, was done using a DCD donor. Over the ensuing five decades, the practice of DCD transplantation had largely been replaced by DBD donation. Unfortunately, the rate of DBD hearts made available for donation has not met the growing demand (22). To help alleviate this shortage, the use of DCD donor hearts resumed in 2014. Early data indicated no difference in operative mortality or survival when using DCD hearts compared to DBD hearts (23, 24).

One of the major factors limiting the use of DCD hearts is the requisite ischemic period compounded by injury that occurs at the time of reperfusion. It has been previously demonstrated through large animal and limited human heart experiments that DCD hearts are recoverable with WITs of up to 30 min (25, 26). As such, over 80% of donated DCD hearts with WIT longer than 30 min are typically unused for the fear of primary graft dysfunction, thereby contributing to the deficiency of hearts for transplant. Previous work by our group has established that beyond 25 min of WIT, DCD rat hearts sustain significant injury and are unable to regain acceptable levels of function following reperfusion (27). Our lab has also studied that administration of CyA or MDL, at the time of reperfusion, significantly reduces infarct size in DCD rat hearts with 25 min of WIT. The present study explores whether co-administration of both CyA and MDL would have synergistic benefit on DCD heart with prolonged WIT (35 min).

DCD hearts sustain injury during both the ischemic period as well as at the time of reperfusion. In addition, it has been established that IRI is proportional to the duration of ischemia. In fact, reperfusion injury can account for up to 50% of the injury sustained by these hearts (11). This is important to note as per the DCD organ procurement stipulations, the reperfusion period is the only time during which interventions may be applied. Much of the injury following reperfusion occurs secondary to mitochondrial damage, specifically the damaged electron transport chain (ETC) as well as degradation of mitochondrial structural proteins (28, 29). While the exact mechanisms behind these events are multifactorial, MPTP opening and CPN1/CPN2 activation are key mediators and each have well-established inhibitors; CyA and MDL, respectively (9, 30, 31).

Our results demonstrate several findings related to the use of CyA and MDL in limiting IRI in DCD rat hearts. First, while the use of CyA or MDL reduced infarct size in rat hearts following 35 min of ischemia compared to untreated hearts, the reduction was more profound in the CyA-treated hearts. CyA reduces cardiac injury by inhibiting MPTP, whereas MDL mitigates injury by protecting structural proteins through inhibition of ubiquitous calpains. We therefore sought to extend this work by combining CyA and MDL to determine whether a synergistic effect could be achieved by targeting different mechanisms. A modest reduction in infarct size was observed in hearts treated with the combination of CyA and MDL compared to MDL alone. It is well established that there is a unique interplay between CyA and its target, Cyclophilin-D (CyP-D), to inhibit MPTP opening at the time of reperfusion (32, 33). In addition, it has been shown that administration of MDL can also decrease MPTP opening in mouse hearts, despite this not being its primary mechanism of action (28). Perhaps sharing the common pathway on MPTP could have negated the synergistic benefits of combined treatment.

What is less known is how these compounds interact with other key mediators of MPTP opening, such as the adenine nucleotide translocase (ANT) and phosphate carrier (PiC). While CyP-D plays a key role in MPTP opening, previous work has shown that even in the absence of CyP-D, or in the presence of CyA, the MPTP can continue to remain open, specifically in environments with higher concentrations of Ca^2+^ or reactive oxygen species (ROS) (34–36), as would be seen in these experiments with prolonged WIT. In these situations, mediators such as ANT or PiC may play a more dominant role even if not essential for MPTP opening and more research into their interactions with CyA and MDL are needed to potentially better understand these observations.

Similar to infarct size, both CyA- and MDL-treated hearts showed improvements in LVDP and RPP compared to untreated controls. Interestingly, hearts treated with the combination of CyA and MDL exhibited the poorest functional performance among all treatment groups. Cardiac function can be influenced by both the extent of myocardial infarction and myocardial stunning, particularly during the acute reperfusion phase. This phenomenon was first reported by Hendrick et al., who demonstrated that reperfused myocardial tissue exhibited diminished contraction following a 15-min period of ischemia, even in the absence of significant infarction. They further noted that the tissue was able to recover function after 48 h (37). Previous work from our lab corroborates these findings, showing that CyA-treated DCD hearts exhibited discordant functional data relative to their reduction in infarct size but were able to recover function 48 h after heterotopic transplantation (17). Given the prolonged WIT used in this study combined with a 90-min reperfusion period, it is reasonable to suggest that these conditions contributed to the observed functional outcomes, and that longer reperfusion periods might allow many of the trends reported to reach statistical significance.

The present study has several limitations. While Langendorff reperfusion is a well-established and widely used method for *ex vivo* reperfusion, it does not impose afterload on the heart as a working heart model would, which could affect the interpretation of functional data. Additionally, although K-H buffer is commonly used as a perfusate for *ex vivo* reperfusion, it does not replicate the chemistry of whole blood, particularly its oxygen-carrying capacity, which may influence infarct size measurements. We also acknowledge that, although this study focuses on the augmentation of MPTP opening and calpain activation, cardiomyocyte injury is a multifactorial process, and other pathways of cell death, such as ferroptosis, should be considered. Future studies investigating the interplay between these pathways and their contribution to cardiac injury would enhance our understanding of ischemia–reperfusion injury. Furthermore, the optimal dose and timing of both CyA and MDL remain unclear. Different dose combinations or variations in the timing of administration could potentially yield more pronounced reductions in infarct size. Finally, while myocardial stunning is a plausible explanation for the discordance between significant infarct size reductions and the lack of proportionate improvements in functional data, this would need to be confirmed using imaging techniques such as MRI or PET, which could be pursued in future studies involving heterotopic transplantation.

## 5 Conclusion

Our study demonstrates that both CyA and MDL, given at the time of reperfusion, significantly reduce infarct size in DCD rat hearts following 35 min of ischemia. In addition, the reduction in infarct size is more profound in CyA-treated hearts compared to MDL-treated hearts. No significant synergistic effect in reducing infarct size was seen with CyA and MDL combination. While not conclusive, these results suggest that MPTP opening may have a more prominent role in the development of IRI over CPN1/CPN2 activation.

## Data Availability

The raw data supporting the conclusions of this article will be made available by the authors, without undue reservation.
